# Application of Machine Learning for Predicting Anastomotic Leakage in Patients with Gastric Adenocarcinoma Who Received Total or Proximal Gastrectomy

**DOI:** 10.3390/jpm11080748

**Published:** 2021-07-29

**Authors:** Shengli Shao, Lu Liu, Yufeng Zhao, Lei Mu, Qiyi Lu, Jichao Qin

**Affiliations:** 1 Department of Surgery, Tongji Hospital, Tongji Medical College, Huazhong University of Science and Technology, Wuhan 430030, China; m201775947@hust.edu.cn (S.S.); luliu048@tjh.tjmu.edu.cn (L.L.); 2012302180379@whu.edu.cn (Y.Z.); mulei100@hust.edu.cn (L.M.); m202076468@hust.edu.cn (Q.L.); 2 Molecular Medicine Center, Tongji Hospital, Tongji Medical College, Huazhong University of Science and Technology, Wuhan 430030, China; 3 Department of Vascular Surgery, First Hospital of Lanzhou University, Lanzhou University, Lanzhou 730030, China

**Keywords:** artificial intelligence, machine learning, anastomotic leakage, gastric adenocarcinoma, total gastrectomy, proximal gastrectomy

## Abstract

Anastomotic leakage is a life-threatening complication in patients with gastric adenocarcinoma who received total or proximal gastrectomy, and there is still no model accurately predicting anastomotic leakage. In this study, we aim to develop a high-performance machine learning tool to predict anastomotic leakage in patients with gastric adenocarcinoma received total or proximal gastrectomy. A total of 1660 cases of gastric adenocarcinoma patients who received total or proximal gastrectomy in a large academic hospital from 1 January 2010 to 31 December 2019 were investigated, and these patients were randomly divided into training and testing sets at a ratio of 8:2. Four machine learning models, such as logistic regression, random forest, support vector machine, and XGBoost, were employed, and 24 clinical preoperative and intraoperative variables were included to develop the predictive model. Regarding the area under the receiver operating characteristic curve (AUC), sensitivity, specificity, positive predictive value (PPV), negative predictive value (NPV), and accuracy, random forest had a favorable performance with an AUC of 0.89, a sensitivity of 81.8% and specificity of 82.2% in the testing set. Moreover, we built a web app based on random forest model to achieve real-time predictions for guiding surgeons’ intraoperative decision making.

## 1. Introduction

Gastric adenocarcinoma is the most common malignancy in the upper gastrointestinal tract, and total and proximal gastrectomy are the two main surgical procedures to remove gastric adenocarcinoma in the proximal two-thirds of the stomach [1]. However, there are serious complications in both procedures, the most serious being anastomotic leakage (AL). The incidence of AL in esophagogastrostomy or esophagojejunostomy varies from 1.7% to 15% [2,3,4]; AL is not only associated with 0% to 50% perioperative mortality but also poor overall survival [4]. Early detection of AL is critical because delayed treatment is associated with higher morbidity and mortality. Identifying high-risk patients of AL is important for guiding the surgeons’ decision making, such as a more rigorous anastomotic operation and placing a jejunal feeding tube. Due to low morbidity, it is difficult to evaluate the risk of AL individually. Although there is ever-increasing knowledge about AL and some studies have attempted to analyze risk factors to build predicting tools, there is still no reported model accurately predicting AL in patients with gastric adenocarcinoma who received total or proximal gastrectomy [5,6,7].

Artificial intelligence has recently shown great potential in various medical fields [8,9]. Machine learning, a subset of artificial intelligence, outperforms other technologies in developing predictive models [10,11]. Machine learning is to “learn” from data without explicit programming, which means that the performance of a specific task improves with experience (i.e., more data and variables). Recently, machine learning has reached encouraging achievements in diagnostic methods, such as the accuracy of the Gastrointestinal Artificial Intelligence Diagnostic System in detecting upper gastrointestinal cancer, which was more than 91.7% [12]. Deep learning models successfully classified microsatellite instability in gastrointestinal cancer [13,14]. In addition, Eiryo et al. developed a model for preoperative diagnostic and prognostic prediction of epithelial ovarian cancer based on peripheral blood biomarkers through machine learning [15]. Although many previous studies have demonstrated the advantages of artificial intelligence in classifying diseases, there are still no models for predicting AL in patients with gastric adenocarcinoma who received total or proximal gastrectomy. In this study, we aimed to develop a diagnostic system using preoperative and intraoperative variables through machine learning algorithms to predict AL in patients with gastric adenocarcinoma who received total or proximal gastrectomy. 

## 2. Materials and Methods

### 2.1. Patients and Variables

Data from 1915 consecutive patients diagnosed with gastric adenocarcinoma who received total or proximal gastrectomy from 1 January 2010 to 31 December 2019 in the Department of Gastrointestinal Surgery, Tongji Hospital, Huazhong University of Science and Technology, were collected. The following 24 variables were included: gender, age, body mass index (BMI), American Society of Anesthesiologists classification score (ASA), previous abdominal surgical history, hypertension, diabetes, Brinkman index (the number of cigarettes smoked per day multiplied by the number of years of smoking), alcohol use, tumorous obstruction, total or proximal gastrectomy, esophagogastrostomy or esophagojejunostomy, combined resection of other organs, type of surgery, operative time, intraoperative blood loss, neoadjuvant chemotherapy or radiotherapy, intraperitoneal chemotherapy, drainage tube, nasogastric tube, preoperative albumin and hemoglobin levels, maximum tumor diameter, and clinical stages. Senior surgeons performed all procedures, and the D2 procedure was adopted as the standard surgical technique. In order to develop the machining learning model, patients with the following factors were excluded: acute complications of the adenocarcinoma such as perforation or bleeding (*n* = 58), palliative excision (R1 or R2, *n* = 52), and missing data (*n* = 145). Finally, 1660 patients were chosen for the study; among them, 525 patients received proximal gastrectomy, and 1135 patients received total gastrectomy. Three authors independently collected all clinical variables and the conflict data were recorded by one of the authors and confirmed through final discussion. 

### 2.2. Outcome

The diagnosis of AL is based on the combination of clinical manifestations and imaging findings. The diagnosis of AL is determined when the passage of gastrointestinal contents from the drainage tube or the oral water-soluble contrast agent leak outside of the gastrointestinal tract. Alternatively, AL can be diagnosed through secondary surgical exploration when the integrity of the anastomosis is interrupted within 30 days after surgery. Case collectors recorded cases with an ambiguous diagnosis of AL, and the classification of these cases was determined during a final discussion by the review team, which comprised two senior gastrointestinal surgeons.

### 2.3. Machine Learning Algorithms 

In this study, four types of machine learning algorithms were assessed: logistic regression (LR), random forest (RF), support vector machine (SVM), and XGBoost. The data were randomly divided into training and testing sets (8:2); the under-sampling method was used to train all algorithms because of the class imbalance of the data. In order to increase the accuracy of the algorithms, simple min-max normalization was used to keep the continuous variables within a range of [0, 1]. The performance of each model was optimized by hyperparameter adjustment. In the testing set, the performance of the machine learning models was evaluated by area under the receiver operating characteristic curve (AUC); the diagnostic ability of the models was verified by calculating sensitivity, specificity, positive predictive value (PPV), negative predictive value (NPV), and accuracy. All machine learning algorithms were implemented using scikit-learn package, version 0.24.1 in Python 3.8.5. A web app was built using the Streamlit package, version 0.78.0 through Spyder 4.2.5.

### 2.4. Statistical Analysis

Continuous variables were shown as mean (SD) and categorical variables as count (%). Student’s *t*-test was used to compare the difference for continuous variables; categorical variables were compared through the Chi-square test. All statistical tests were two-tailed, and *p* < 0.05 is considered a statistically significant difference. Confidence intervals (CIs) of sensitivity, specificity, PPV, NPV, and accuracy were calculated using Clopper—Pearson method. The above analyses were performed in IBM SPSS 24.0 (SPSS for Windows, IBM Corporation, Armonk, NY, USA) or VassarStats (online).

## 3. Results

### 3.1. Summary of Demographic and Clinical Characteristics for Training and Testing Sets

The study included 1660 patients, and the incidence of AL was 2.17% (36/1660). In order to develop the machine learning model, 1328 cases were assigned to the training set, and the remaining 332 cases were assigned to the testing set. A comparison of the training set and the testing set are shown in Table 1. In the training set, 31.9% of the patients received esophagogastrostomy, compared with 26.8% in the testing set. Total gastrectomy was performed in 67.6% of cases in the training set and 71.4% of cases in the testing set. The incidence of AL was 1.9% (25/1328) of cases in the training set and 3.3% (11/332) of cases in the testing set.

### 3.2. Performance of the Machine Learning Algorithms

**Figure 1:**
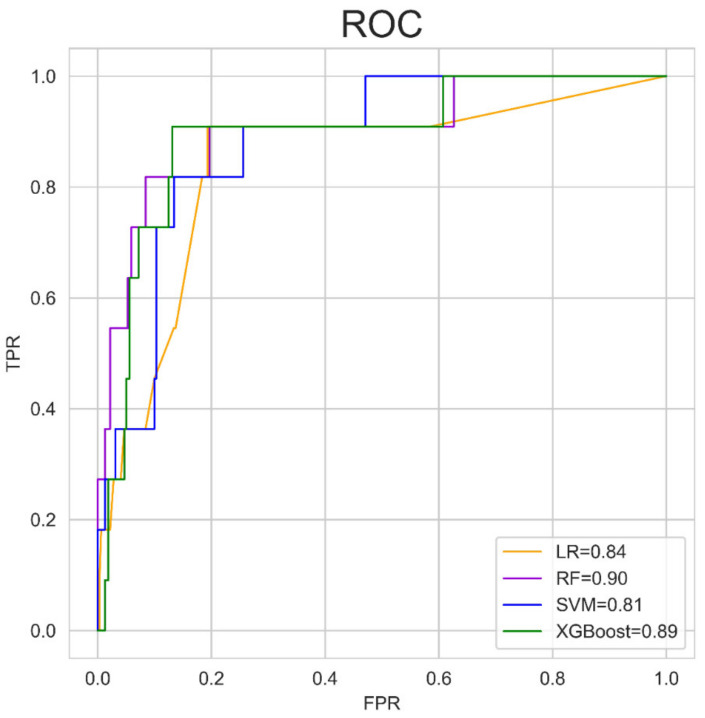
Performance of the machine learning algorithms for predicting AL in the testing set. ROC: receiver operating characteristic curve; LR: logistic regression; RF: random forest; SVM: support vector machine; TPR: true positive rate; FPR, false positive rate.

We evaluated the predictive performance of four machine learning algorithms in the testing set by AUC. The data indicated that RF and XGBoost had better predictive performance (RF-AUC = 0.90, XGBoost-AUC = 0.89), whereas SVM performed poorly (SVM-AUC = 0.81) (Figure 1). Notably, RF and XGBoost are ensemble classifiers based on weak classifiers. The predictive results of each machine model in the testing set are shown in Table 2.

### 3.3. Predictive Abilities of the Machine Learning Models

Five indicators were used to calculate the machine learning models’ predictions in the testing set. The results indicated that RF model performed with higher specificity (0.822 (0.775–0.862) vs. 0.701 (0.647–0.750), *p* < 0.001) and accuracy (0.822 (0.776–0.861) vs. 0.708 (0.656–0.756), *p* < 0.001) than SVM. Moreover, when compared with XGBoost, RF model also had higher specificity (0.822 (0.775–0.862) vs. 0.723 (0.670–0.770), *p* = 0.003) and accuracy (0.822 (0.776–0.861) vs. 0.729 (0.678–0.776), *p* = 0.004), no statistical difference was observed between LR and RF in the five indicators (Table 3). To make the model more clinically practical, we developed an online app (https://gasal.21cloudbox.com/ (available from 14 May 2021 to 14 May 2024)) based on the RF model, which allows us to calculate the risk of AL in real-time according to 24 clinical variables from the preoperative and intraoperative periods.

### 3.4. Feature Importance Analysis

**Figure 2:**
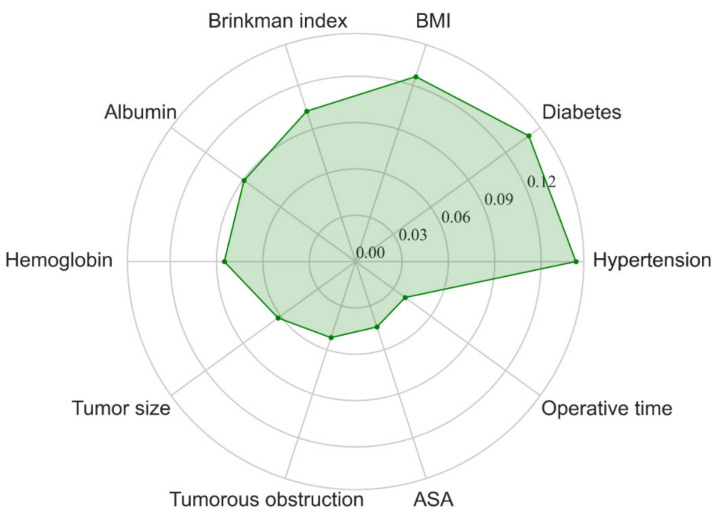
Radar plot for the ten most important variables in predicting AL of the RF model. BMI, body mass index; ASA, American Society of Anesthesiologists classification score; RF, random forest.

To our best knowledge, the occurrence of AL is a result of the interaction of all the relative factors. In order to gain insight into the contribution of each clinical variable to AL, the importance of each clinical variable was calculated through feature importance analysis, and the results showed that hypertension, diabetes, BMI, Brinkman index, albumin, hemoglobin, tumor size, tumorous obstruction, ASA score, and operation time were the ten most important features in the RF model (Figure 2).

## 4. Discussion

AL of esophagogastrostomy or esophagojejunostomy is a serious and life-threatening complication in patients with gastric cancer who received total or proximal gastrectomy. Once AL is diagnosed, continuous parenteral nutrition is a necessary treatment for fasting and gastrointestinal decompression, even though it increases the incidence of related complications. In addition, secondary surgery is required to establish smooth drainage of the leakage and indwelling a jejunal nutrition tube to support enteral nutrition for serious AL. Hence, preoperative or intraoperative identification of high-risk patients with AL may assist intraoperative decision making, such as establishing smooth drainage of the anastomotic site and placing a jejunal feeding tube.

Although the rigorous anastomotic operation is an essential measure in preventing AL, the heterogeneity of individual patients also plays an important role in the occurrence of AL. Most clinicians are familiar with the risk factors of AL, such as anemia, prognostic nutritional index, cardiovascular disease, obesity, and smoking [4,16]. However, it is rare for each patient to have all the risk factors, and these risk factors may have different contributions to the development of AL. Thus, accurately calculating the risk of AL for individual patients has always been a great challenge for surgeons. In order to overcome this difficulty, several attempts have been made to develop prediction models of AL through binary logistic regression analysis. For example, Tu RH et al. proposed a nomogram based on independent risk factors, including age, hemoglobin, and malnourishment, but the model was not validated, and the performance of the model was poor (c-index = 0.675) [5]. Additionally, Chikara Kunisaki et al. also developed a model based on independent risk factors; the data suggested that the model failed to accurately predict AL (AUC = 0.658) [17]. Binary logistic regression analysis is frequently used in analyzing independent risk factors and modeling, which weighs the independent risk factors and generates a linear formula to achieve predictions. Due to the complexity of clinical data distribution, such as multi-dimensional and non-linearly related variables [18], it is difficult for binary logistic regression analysis to generate a high-performance model. In recent years, the global enthusiasm for machine learning technology based on artificial intelligence seems exponential, and machine learning has achieved impressive results due to improvements in computing power. Some evidence shows that machine learning outperforms statistical models [19,20,21,22]. In the realm of precision medicine, which emphasizes personalized treatment, traditional guidelines or a clinicians’ experience can no longer meet the needs of medical decision making. Machine learning, an innovative tool, may meet the needs of precision medicine and select the best treatment strategy for different individual patients. Therefore, we applied machine learning algorithms that do not depend on independent risk factors to develop a predictive model for individual decision making. 

In this study, we investigated 1660 cases of gastric adenocarcinoma patients who received total or proximal gastrectomy in the past 10 years and found that the incidence of AL was 2.17% (36/1660), which similar to previous reports [23,24]. In order to gain a high-performance tool, we applied four machine learning algorithms and found that RF produced the largest AUC and higher specificity and accuracy compared with SVM and XGBoost. To better satisfy the needs of clinicians, we designed a web app based on RF (81.8% sensitivity, 82.2% specificity, and 0.90 AUC) for achieving real-time predictions online. In order to explore the contribution of each variable to the development of AL, feature importance analysis was performed, and the data suggested that hypertension, diabetes, BMI, Brinkman index, albumin, hemoglobin, tumor size, tumorous obstruction, ASA score, and operation time were the ten most important features. Many of these features have been previously reported as important factors in the development of AL [5,25,26,27,28,29,30]. RF is an ensemble learning algorithm that showed great capability in regression and classification tasks and widely applied in medical modeling and feature importance analysis. For example, Tien S Dong et al. employed the RF algorithm to train a predictive model by identifying factors significantly associated with the presence of esophageal varices. They found that the AUC of the model in the validation set was 0.75 [31]. In addition, Chieh-Chen Wu et al. developed a model based on the RF algorithm to predict fatty liver disease using 577 patients’ data and the model’s performance was favorable (AUC = 0.925) [32]. Hence, there is great potential in using RF to develop high-performance models. To our best knowledge, this is the first study to apply a machine learning model, which was developed through clinical preoperative and intraoperative variables to predict AL in patients with gastric adenocarcinoma who received total or proximal gastrectomy. 

There are several limitations to this study. First, this is a retrospective study based on a single center and selection bias, which is difficult to completely avoid. In addition, data from the tension and blood supply of the anastomosis could not be collected in the present study. However, both factors may play important roles in developing AL. Second, we retrospectively analyzed medical records for 10 years, which is not a short period. It is difficult to assess how advancements in medical technology contribute to decreasing AL. Third, the sensitivity of the model at 95% CI is too wide, and the cases diagnosed by the machine learning model for low risk of AL must be further evaluated. Fourth, the model needs external validation. To overcome these limitations, we intend to conduct a further multicenter study.

## 5. Conclusions

In conclusion, based on clinical preoperative and intraoperative variables, a high-performance machine learning model was developed, which may be helpful to surgeons by identifying patients with a high risk of AL, guiding surgeons in intraoperative decision making, and improving perioperative management for the patients. Most importantly, an online app (https://gasal.21cloudbox.com/ (available from 14 May 2021 to 14 May 2024)) was built to meet the needs of further investigations such as the multicenter validation and prospective study. Applying this app can help predict the risk of AL in patients with gastric adenocarcinoma who received total or proximal gastrectomy in a real-time manner.

## Figures and Tables

**Table 1:** Comparison of the training and testing sets.

Variables	Training Set (*n* = 1328)	Testing Set (*n* = 332)	*p* Value
Male, *n* (%)	983 (74.0%)	242 (72.9%)	0.626
Age, mean (SD), years	58.94 (9.80)	59.66 (10.71)	0.242
BMI, mean (SD), kg/m^2^	21.01 (2.65)	21.02 (2.76)	0.930
Hypertension, *n* (%)	312 (23.5%)	67 (20.2%)	0.214
Diabetes, *n* (%)	88 (6.6%)	23 (6.9%)	0.807
Previous abdominal surgery, *n* (%)	260 (19.6%)	60 (18.1%)	0.586
Brinkman index, mean (SD)	221.34 (412.59)	199.94 (316.26)	0.388
Alcohol use, *n* (%)	272 (20.5%)	64 (19.3%)	0.648
Hemoglobin, mean (SD), g/L	119.96 (22.17)	120.24 (23.73)	0.839
Albumin, mean (SD), g/L	38.46 (4.57)	38.74 (4.90)	0.325
Tumor size, mean (SD), cm	4.34 (2.33)	4.36 (2.40)	0.881
Tumorous obstruction, *n* (%)	226 (17.0%)	58 (17.5%)	0.871
Neoadjuvant, *n* (%)	33 (2.5%)	7 (2.1%)	0.842
Total gastrectomy, *n* (%)	898 (67.6%)	237 (71.4%)	0.210
Esophagogastrostomy, *n* (%)	424 (31.9%)	89 (26.8%)	0.073
Combined resection, *n* (%)	68 (5.1%)	21 (6.3%)	0.413
Laparoscopic surgery, *n* (%)	1133 (85.3%)	283 (85.2%)	1.000
Blood loss, mean (SD), ml	146.95 (252.80)	140.66 (222.05)	0.678
Intraperitoneal chemotherapy, *n* (%)	979 (73.7%)	254 (76.5%)	0.326
Nasogastric tube, *n* (%)	1305 (98.3%)	323 (97.3%)	0.263
Indwelling drainage tube, *n* (%)	1317 (99.2%)	325 (97.9%)	0.068
Operative time, mean (SD), minutes	304.28 (60.71)	312.17 (63.11)	0.036
ASA			0.083
1	205 (15.4%)	48 (14.5%)	
2	971 (73.1%)	233 (70.2%)	
3	145 (10.9%)	51 (15.4%)	
4	7 (0.5%)	0 (0.0%)	
Clinical stages			0.353
1	200 (15.0%)	49 (14.8%)	
2	454 (34.2%)	108 (32.5%)	
3	608 (45.8%)	150 (45.2%)	
4	66 (5.0%)	25 (7.5%)	
AL	25 (1.9%)	11 (3.3%)	0.087

SD, standard deviation; BMI, body mass index; ASA, American Society of Anesthesiologists score; AL, anastomotic leakage.

**Table 2:** Predictive results of four machine learning models in the testing set.

Predictions	True Label	
	Cases with AL	Cases without AL
LR		
AL(+)	10	74
AL(−)	1	247
RF		
AL(+)	9	57
AL(−)	2	264
SVM		
AL(+)	10	96
AL(−)	1	225
XGBoost		
AL(+)	10	89
AL(−)	1	232

AL, anastomotic leakage; LR, logistic regression; RF, random forest; SVM, support vector machine.

**Table 3:** Performance of machine learning models in the testing set.

	RF	LR	SVM	XGBoost	*p* Valve (RF vs.)		
					LR	SVM	XGBoost
Sensitivity (95% CI)	0.818 (0.478–0.968)	0.909 (0.572–0.995)	0.909 (0.572–0.995)	0.909 (0.572–0.995)	0.534	0.534	0.534
Specificity (95% CI)	0.822 (0.775–0.862)	0.770 (0.719–0.814)	0.701 (0.647–0.750)	0.723 (0.670–0.770)	0.096	<0.001	0.003
PPV (95% CI)	0.137 (0.068–0.248)	0.119(0.062–0.212)	0.094 (0.049–0.171)	0.101 (0.052–0.182)	0.752	0.392	0.486
NPV (95% CI)	0.992 (0.970–0.999)	0.996 (0.974–1.000)	0.996 (0.972–1.000)	0.996 (0.973–1.000)	0.978	0.981	0.98
Accuracy (95% CI)	0.822 (0.776–0.861)	0.774(0.725–0.818)	0.708 (0.656–0.756)	0.729 (0.678–0.776)	0.122	<0.001	0.004

CI: confidence interval; PPV, positive predictive value; NPV, negative predictive value; LR, logistic regression; RF, random forest; SVM, support vector machine.

## Data Availability

The data used in the present study is available from the corresponding author on reasonable request.
